# Supplementary material for: Biosensor Based Immunoassay: A New Approach for Serotyping of Toxoplasma gondii
Source: Nanomaterials (Basel). 2021 Aug 14;11(8):2065. doi: 10.3390/nano11082065 (PMC8401488; doi:10.3390/nano11082065)
Supplement: Supplementary file 1 [file nanomaterials-11-02065-s001.zip › nanomaterials-1300560-supplementary.pdf]

Supporting Information

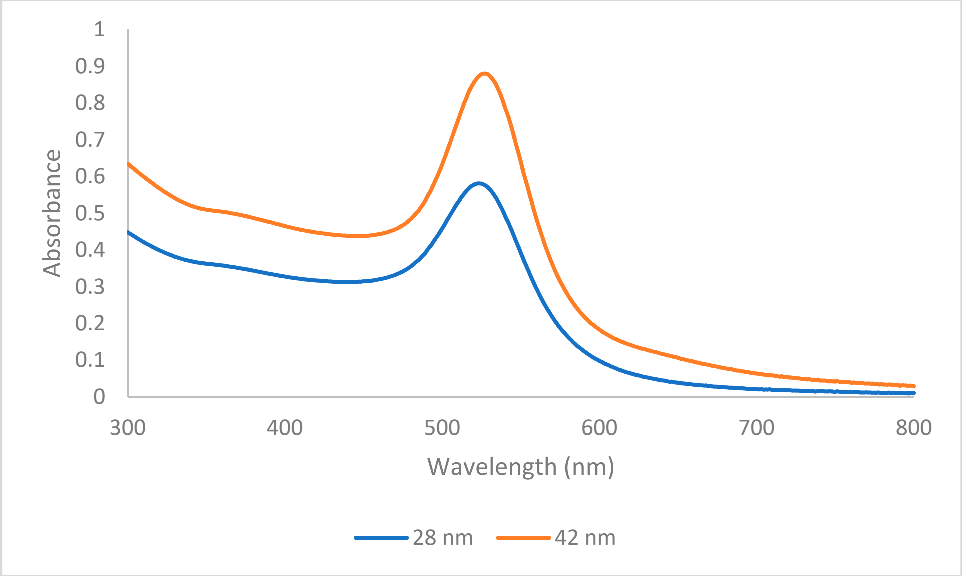

(A)

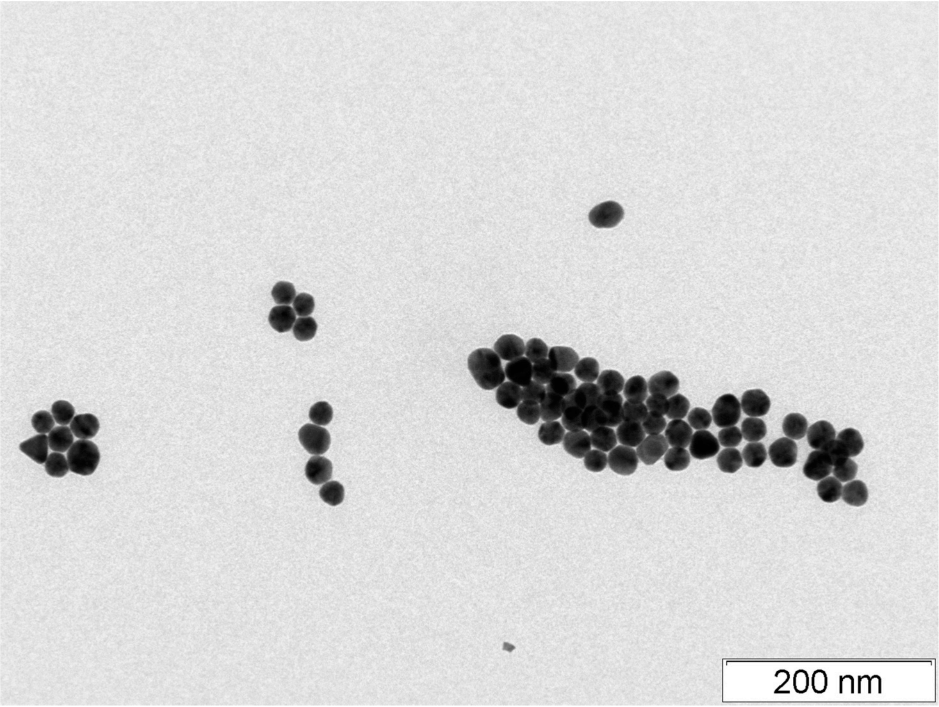

(B)

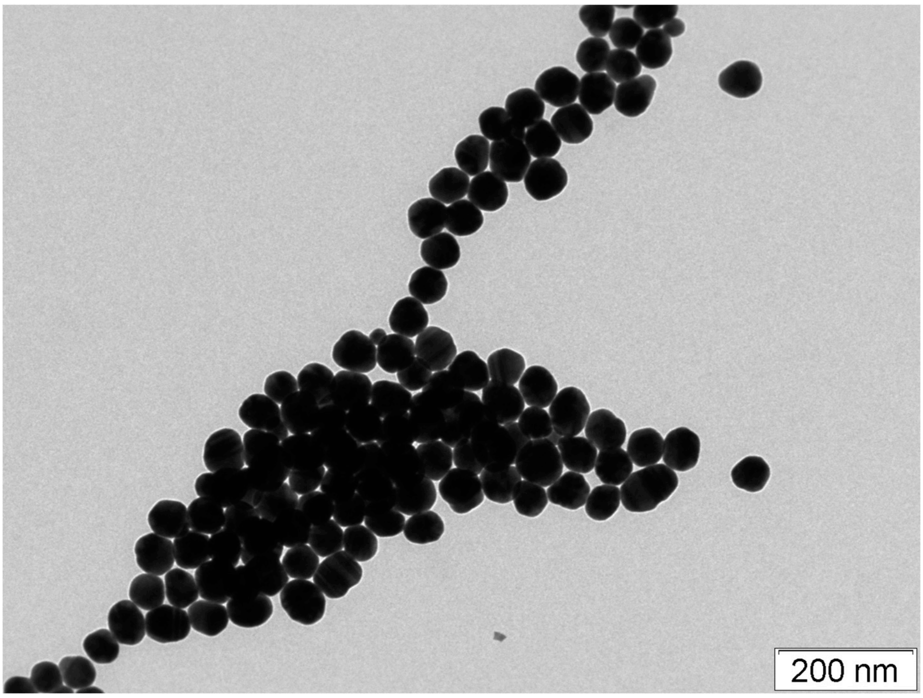

(C)

Figure S1. – UV-Vis spectrum for 28 nm and 42 nm AuNPs (A). TEM images for 28 nm (B) and 42 nm (C) AuNPs.

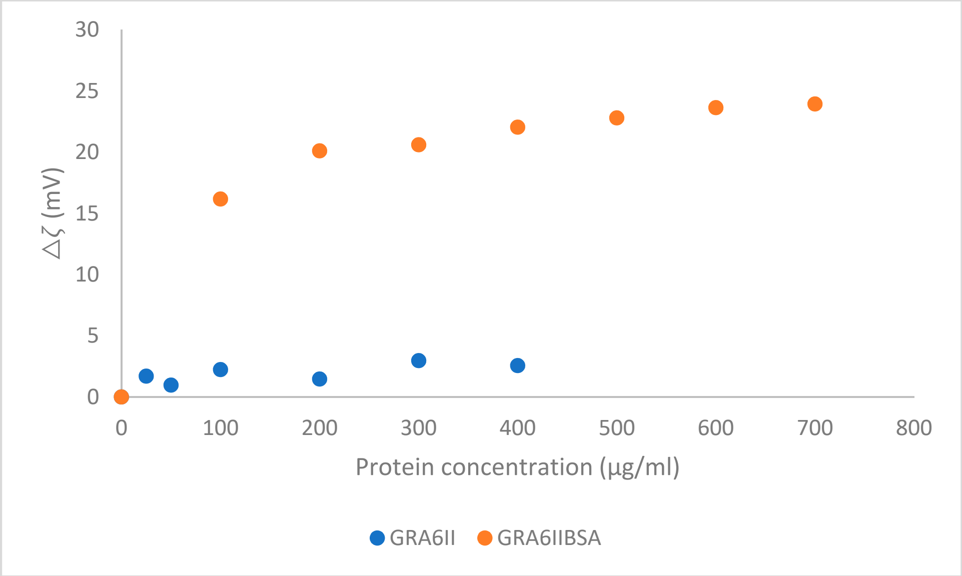

Figure S2. - Variation of zeta potential of AuNPs with increasing concentration of GRA6IIBSA and AuNPs MUA with increasing concentration of GRA6II was measured by ELS.

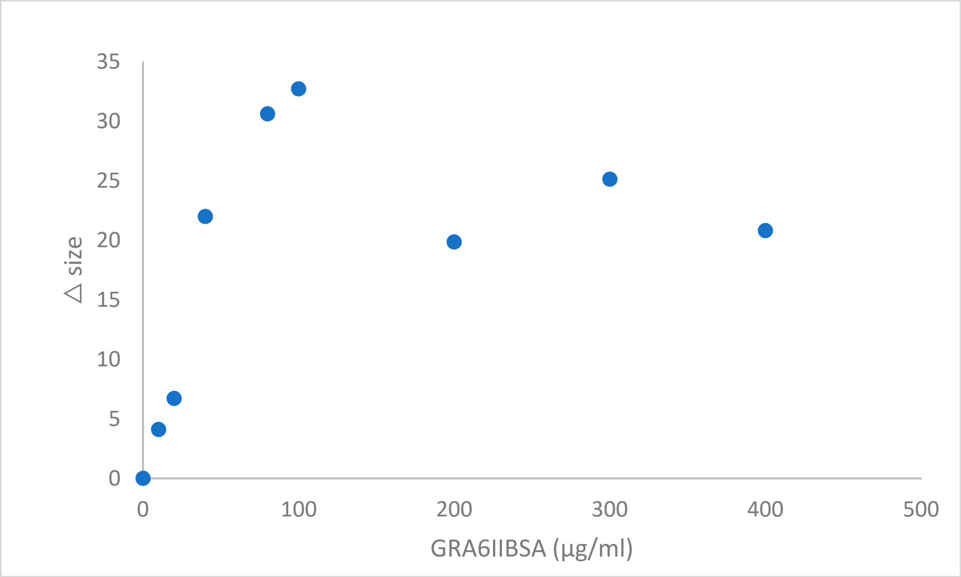

(A)

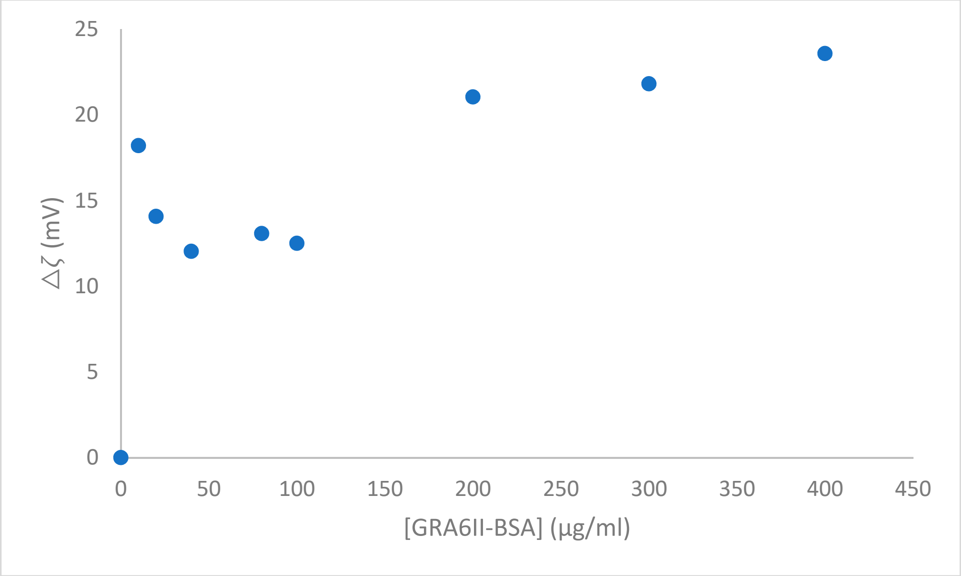

(B)

Figure S3. – 28nm AuNPs-GRA6II bioconjugates were study by DLS and ELS. Variation of hydrodynamic diameter with increasing concentration of peptide was measured by DLS (A). Variation of zeta potential with increasing concentration of peptide was measured by ELS (B).

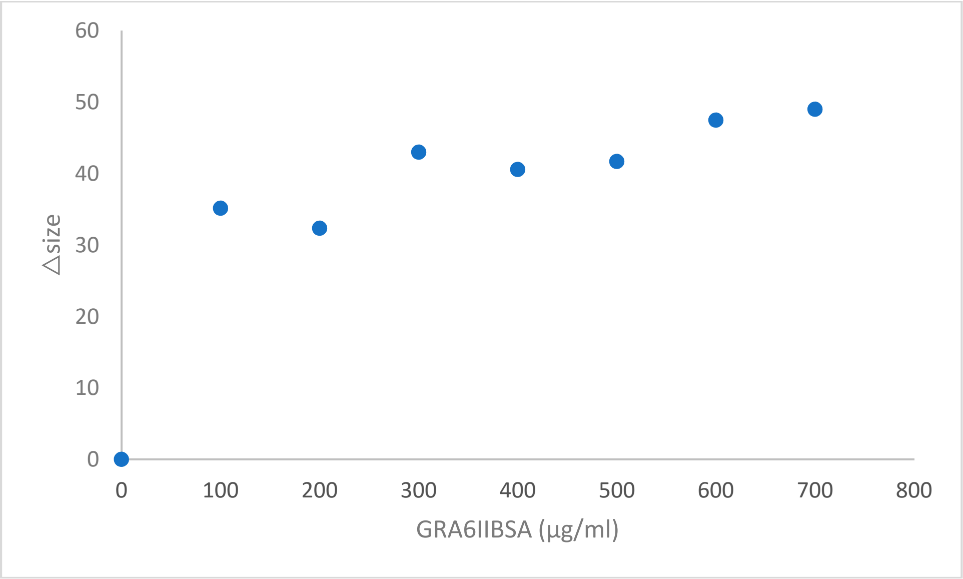

(A)

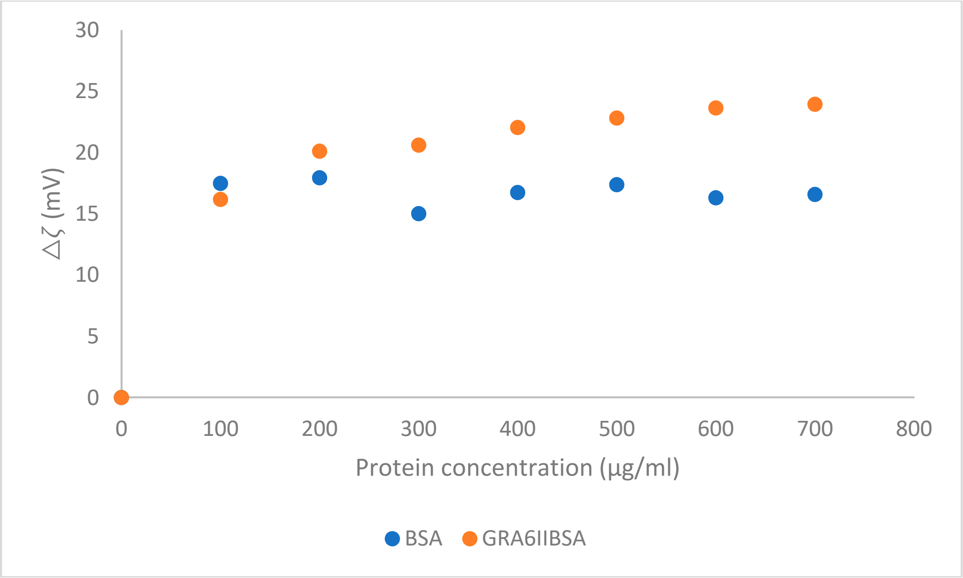

(B)

Figure S4. – 42nm AuNPs-GRA6II bioconjugates were study by DLS and ELS. Variation of hydrodynamic diameter with increasing concentration of peptide was measured by DLS (A). Variation of zeta potential with increasing concentration of GRA6IIBSA and BSA alone was measured by ELS (B).

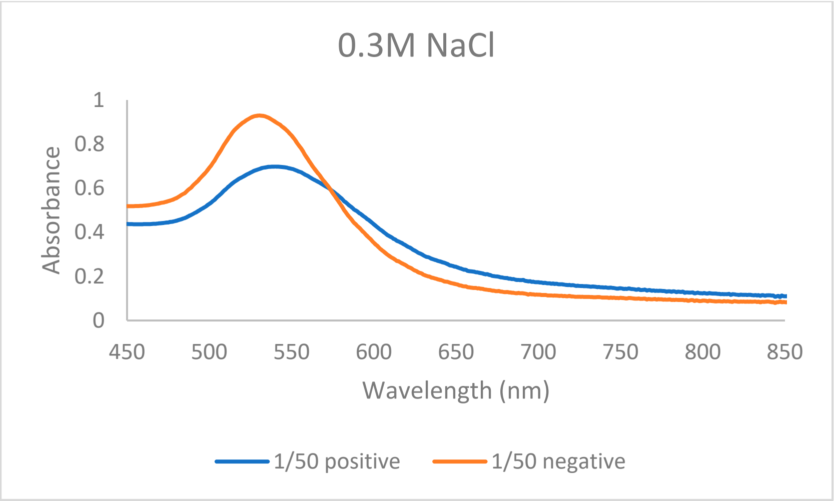

(A)

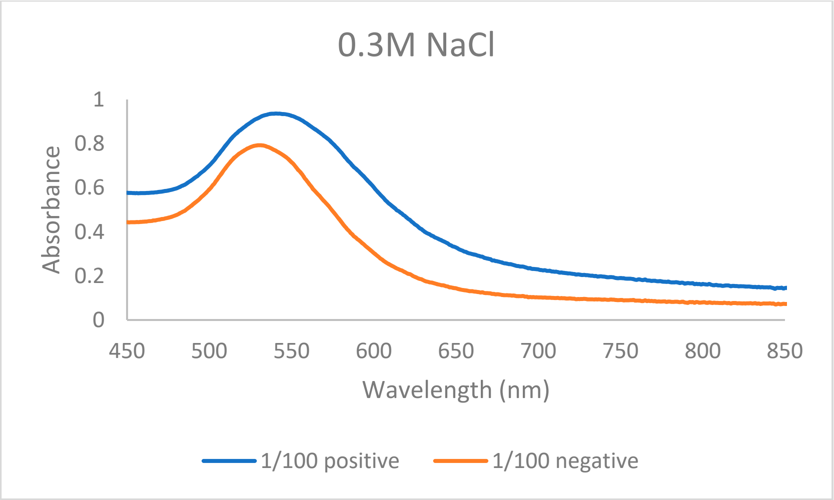

(B)

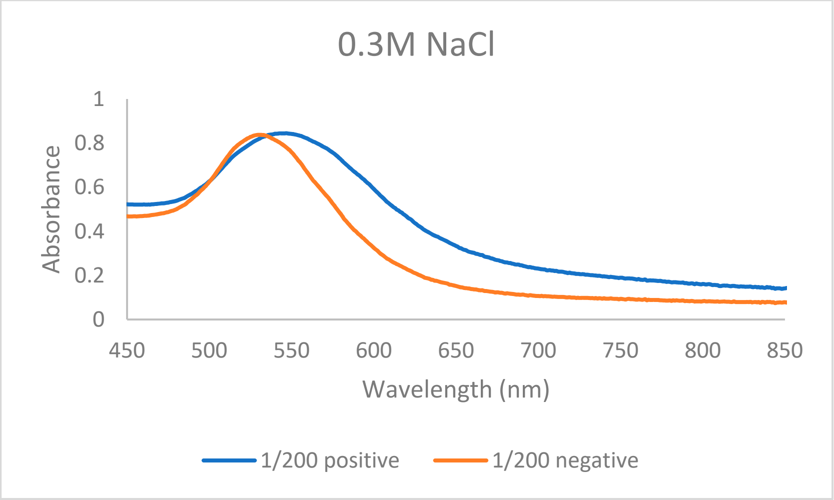

(C)

Figure S5. 28nm AuNPs bioconjugated with GRA6II-BSA in the presence of positive and negative serum sample Three sera dilutions were tested, 1/50 (A), 1/100 (B) and 1/200 (C). UV-Vis spectrum after incubation with NaCl 0.3M.

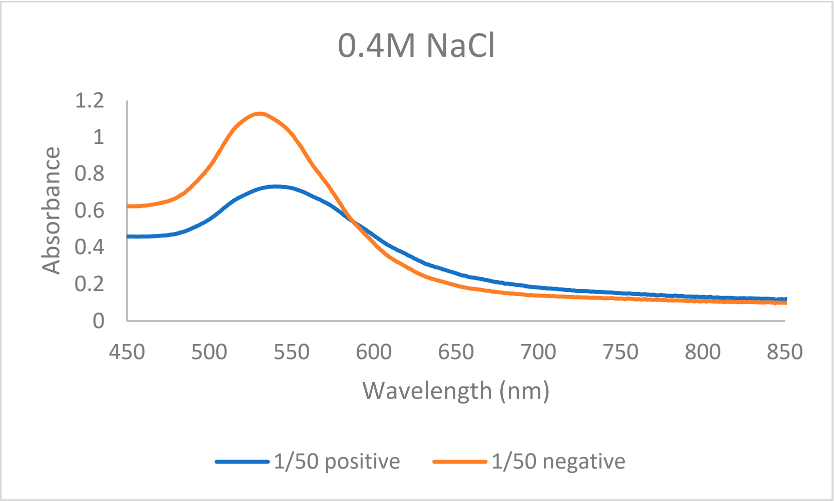

(A)

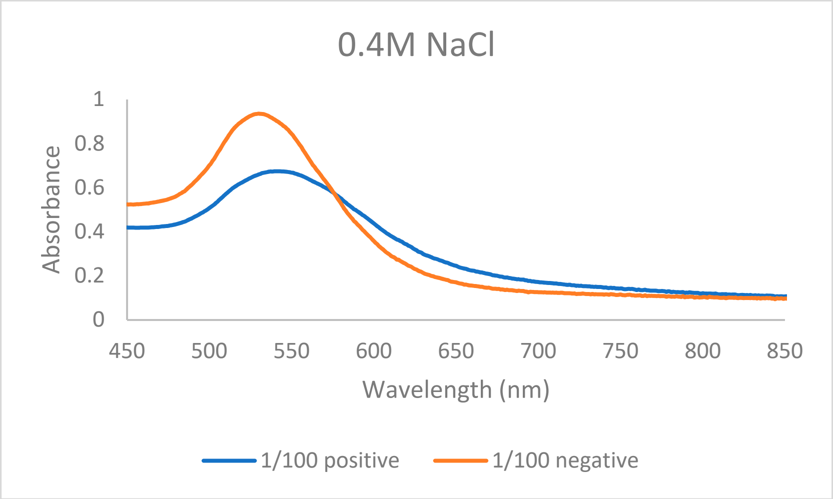

(B)

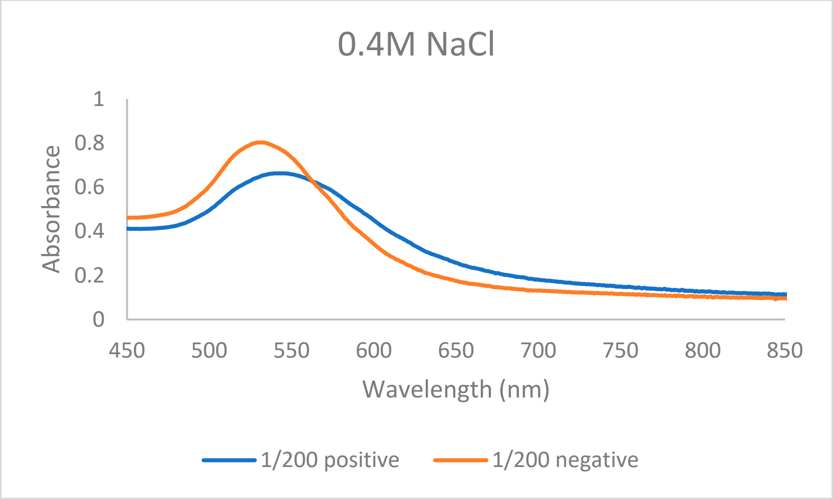

(C)

Figure S6. 28nm AuNPs bioconjugated with GRA6II-BSA in the presence of positive and negative serum sample Three sera dilutions were tested, 1/50 (A), 1/100 (B) and 1/200 (C). UV-Vis spectrum after incubation with NaCl 0.4M.

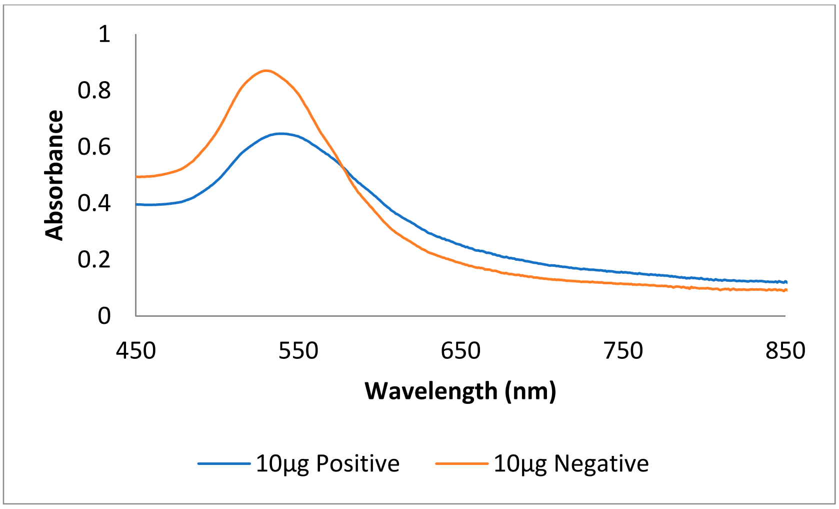

(A)

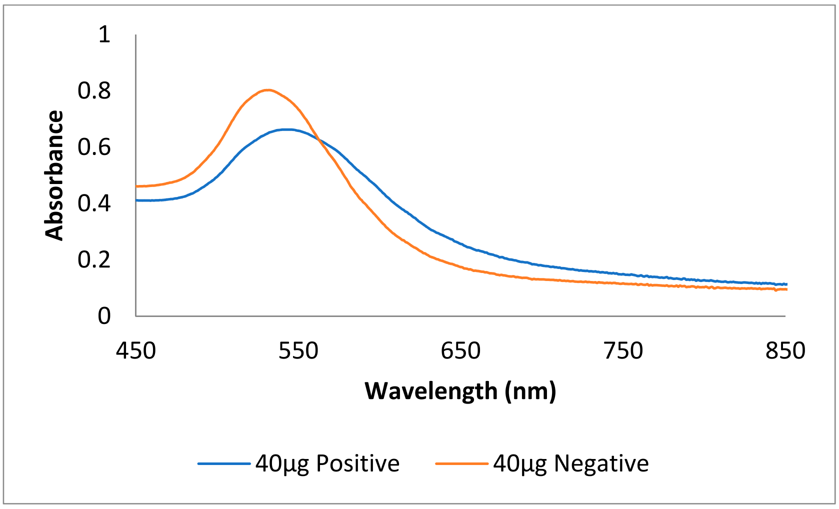

(B)

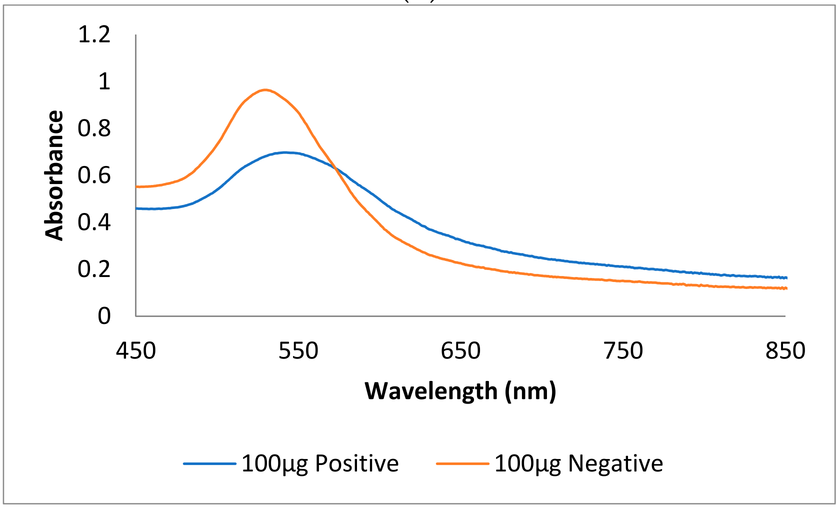

(C)

Figure S7. UV-Vis spectrum of 28nm AuNPs bioconjugated with three GRA6II-BSA concentrations: 10 µg/ml (A), 40 µg/ml (B), and 100 µg/ml (C). Positive and negative sera dilution was 1/200 and NaCl concentration was 0.3M.

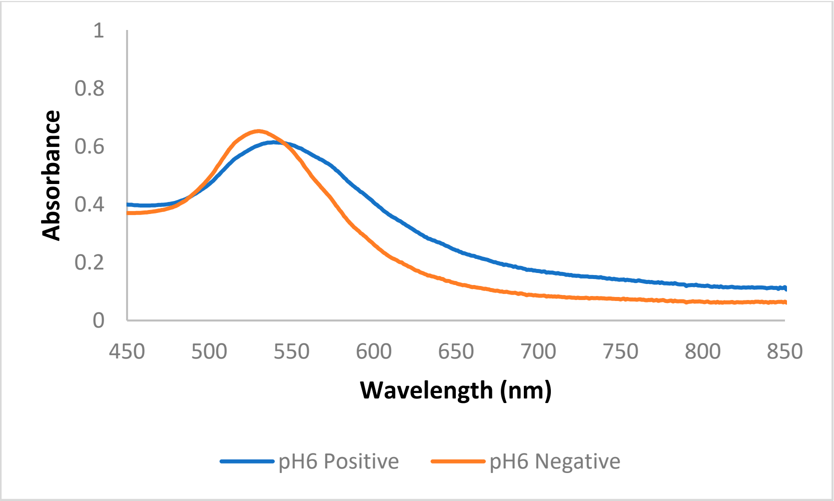

(A)

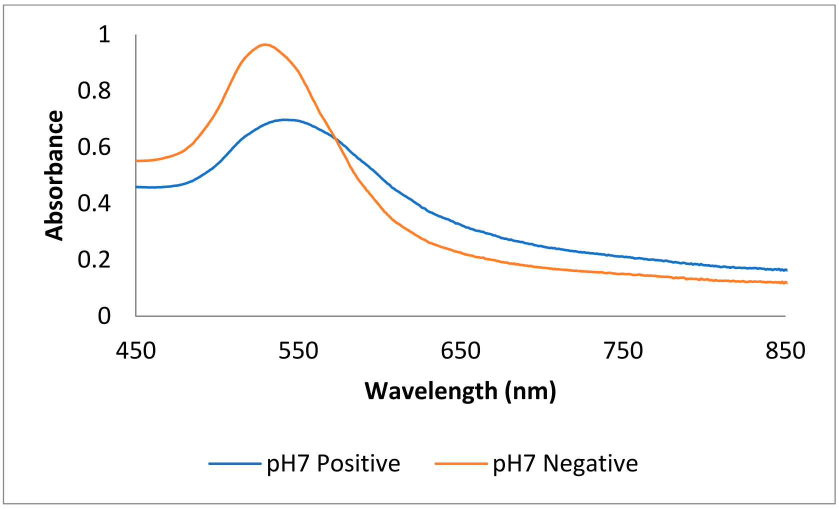

(B)

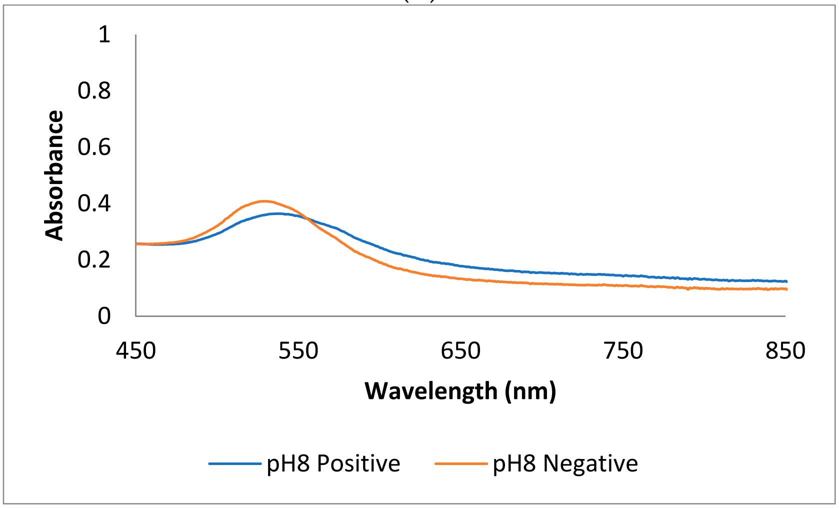

(C)

Figure S8. UV-Vis spectrum of 28nm AuNPs bioconjugated with GRA6IIBSA. Three pH values were compared: pH 6.0 (A), pH7.0 (B) and pH 8.0 (C). Positive and negative sera dilution was 1/200 and NaCl concentration was 0.3M.

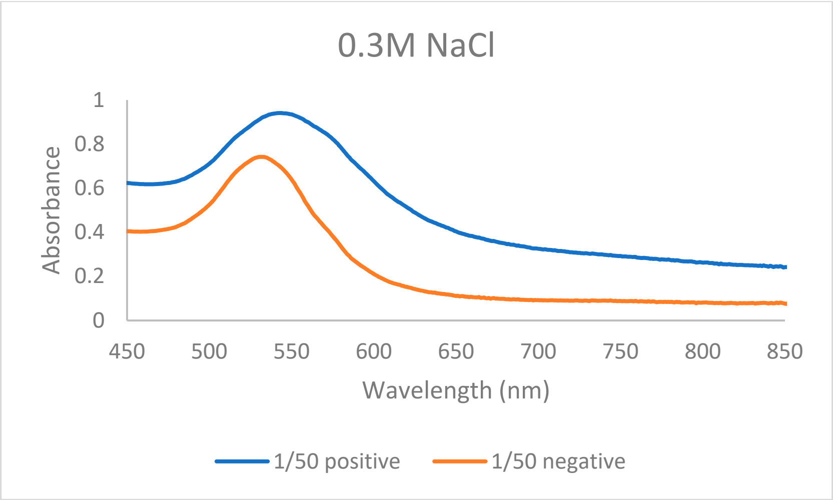

(A)

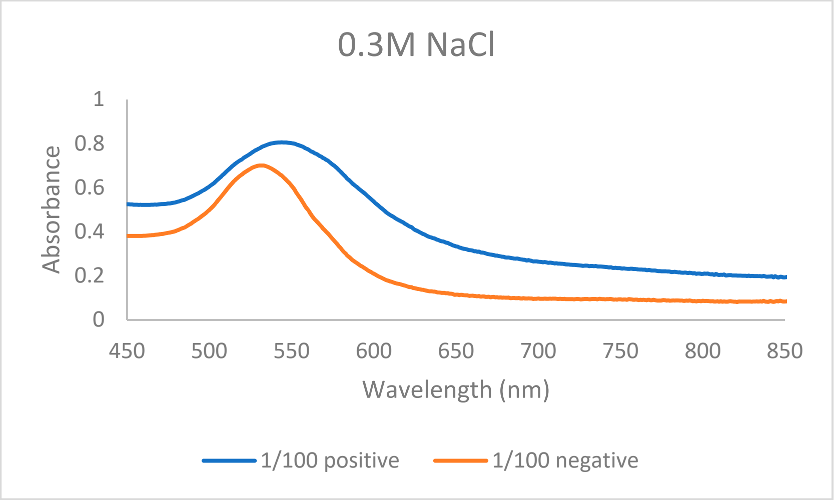

(B)

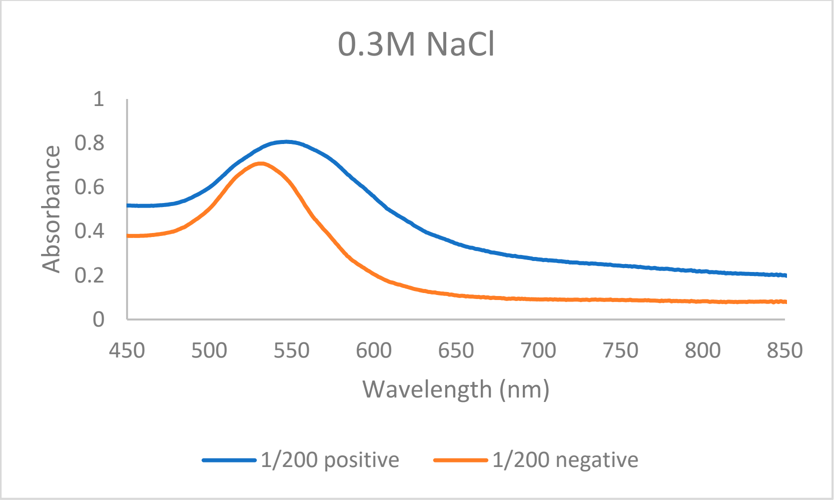

(C)

Figure S9. 42nm AuNPs bioconjugated with GRA6II-BSA in the presence of positive and negative serum sample Three sera dilutions were tested 1/50 (A), 1/100 (B) and 1/200 (C). UV-Vis spectrum after incubation with NaCl 0.3M.

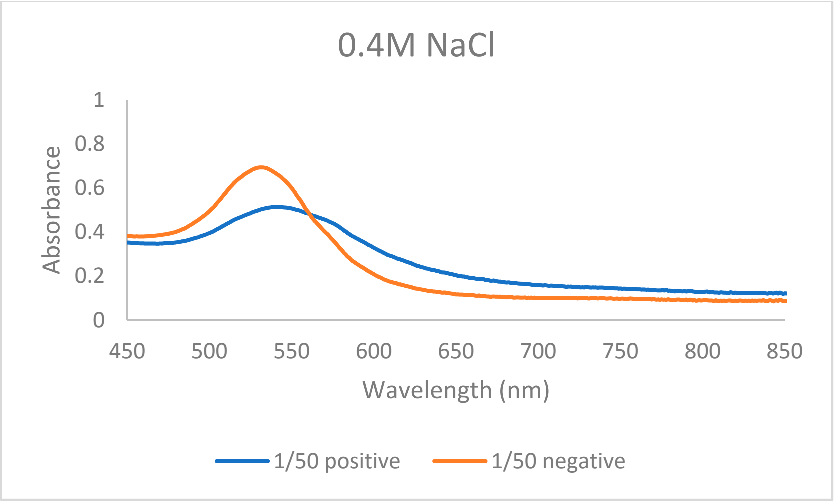

(A)

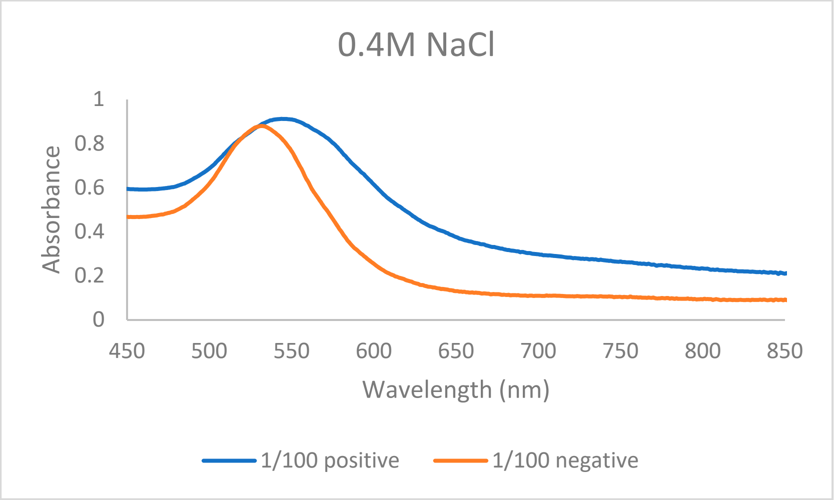

(B)

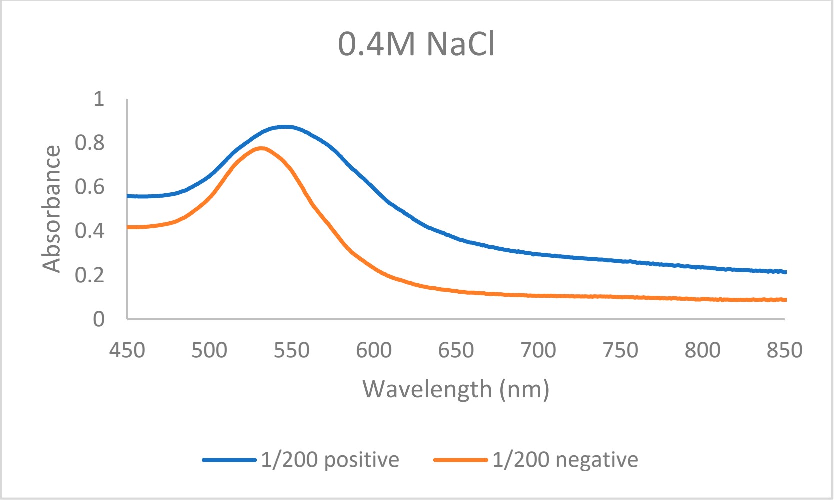

(C)

Figure S10. 42nm AuNPs bioconjugated with GRA6II-BSA in the presence of positive and negative serum sample Three sera dilutions were tested 1/50 (A), 1/100 (B) and 1/200 (C). UV-Vis spectrum after incubation with NaCl 0.4M.

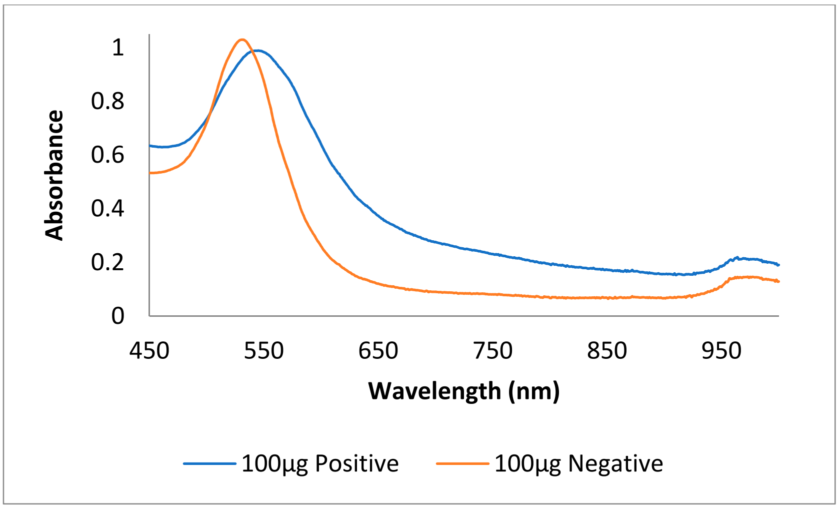

(A)

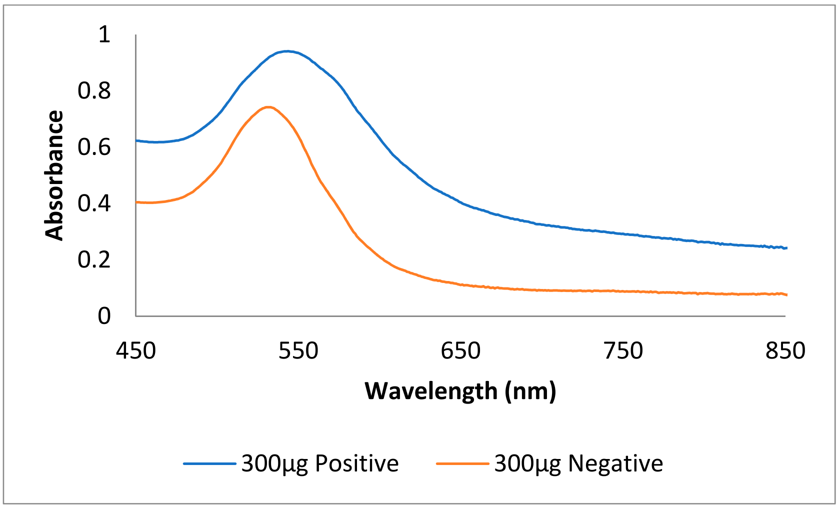

(B)

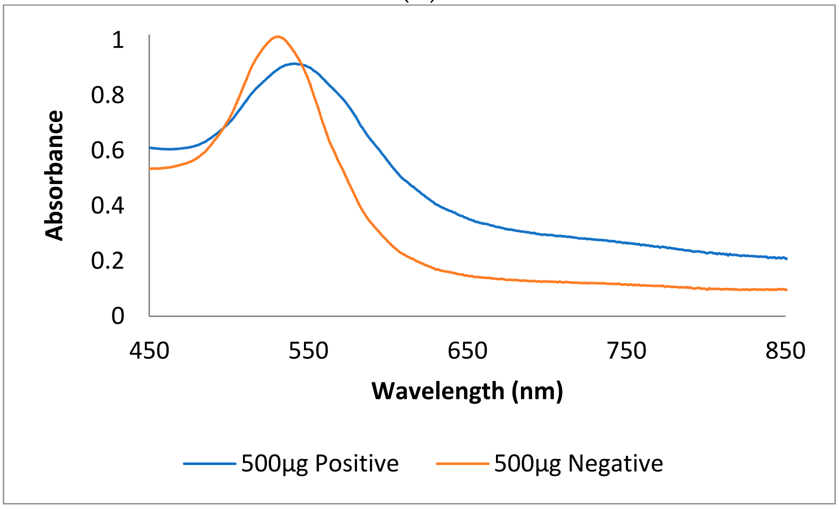

(C)

Figure S11. UV-Vis spectrum of 42nm AuNPs bioconjugated with three GRA6II-BSA concentrations: 100 µg/ml (A), 300 µg/ml (B), and 500 µg/ml (C). Positive and negative sera dilution was 1/50 and NaCl concentration was 0.3M.

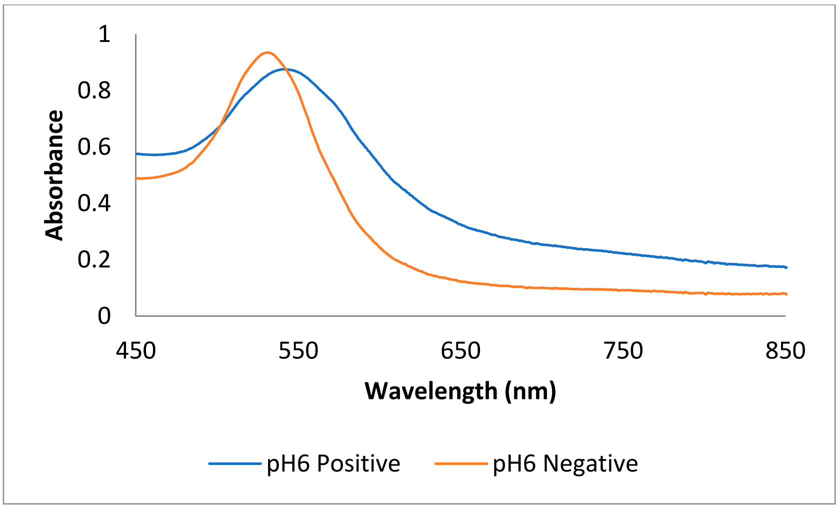

(A)

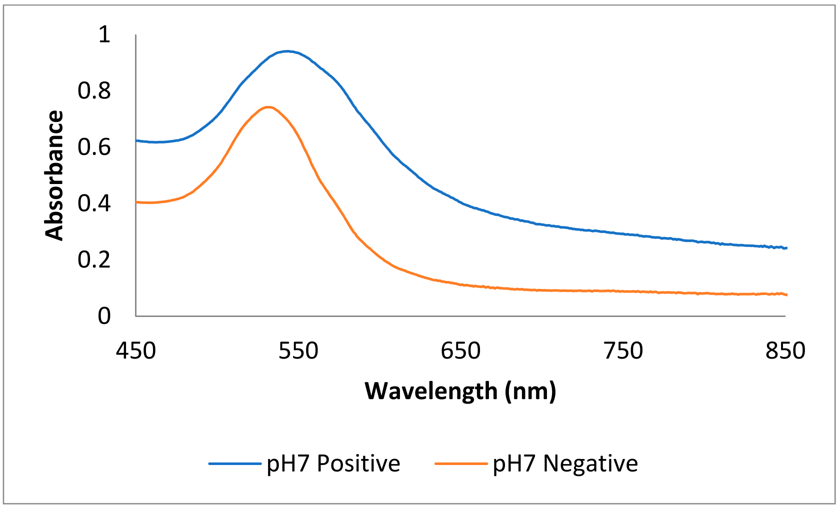

(B)

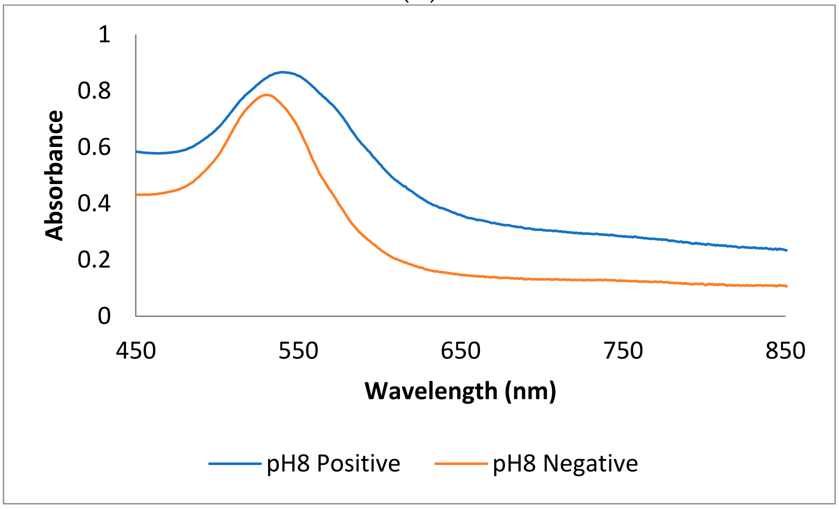

(C)

Figure S12. UV-Vis spectrum of 42nm AuNPs bioconjugated with GRA6IIBSA. Three pH values were compared: pH 6.0 (A), pH7.0 (B) and pH 8.0 (C). Positive and negative sera dilution was 1/50 and NaCl concentration was 0.3M.

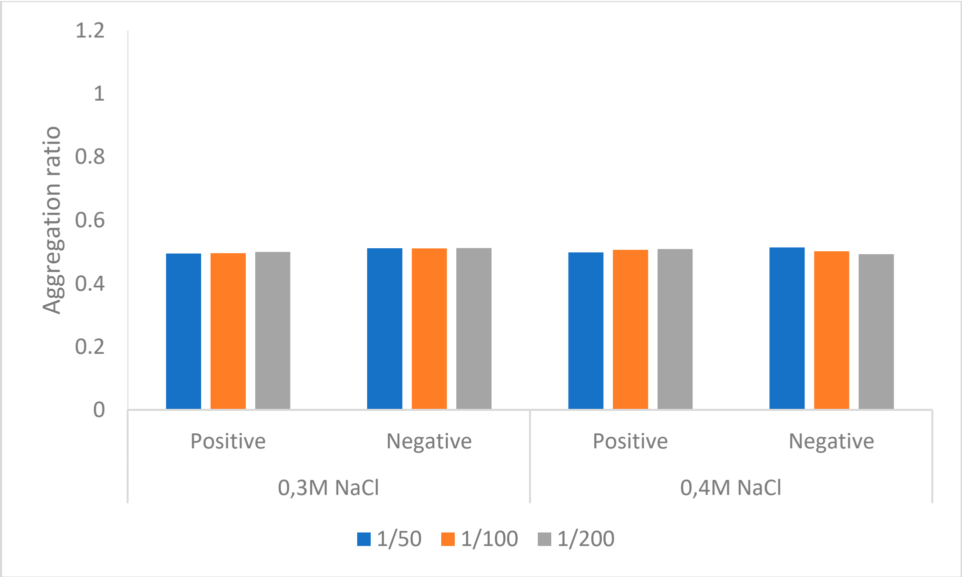

Figure S13. – 42nm AuNPs bioconjugated with BSA in the presence of positive and negative serum samples. Three sera dilutions were tested (1/50, 1/100 and 1/200) and two NaCl concentrations (0.3M and 0.4M).

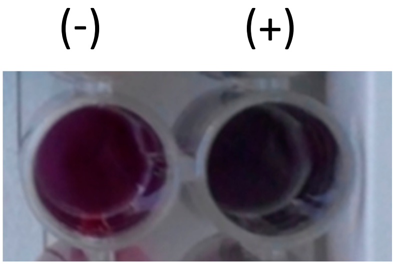

(A)

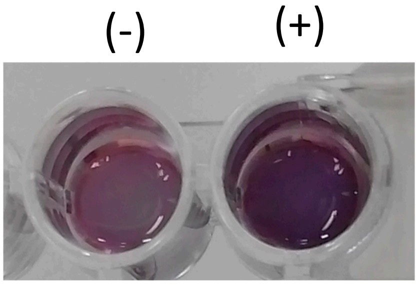

(B)

Figure S14. – Tween 20 (A) and ethanolamine (B) were used as blocking agents. Both reagents induced AuNPs-GRA6II aggregation in the presence of positive and negative serum samples.

(A)

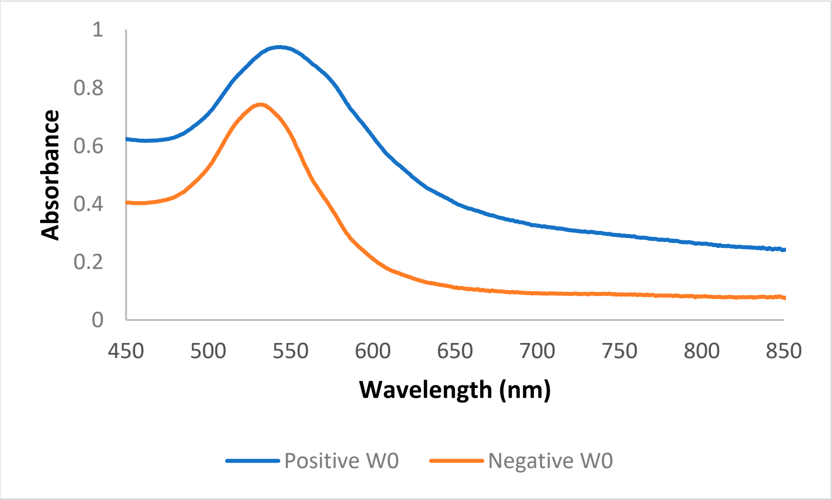

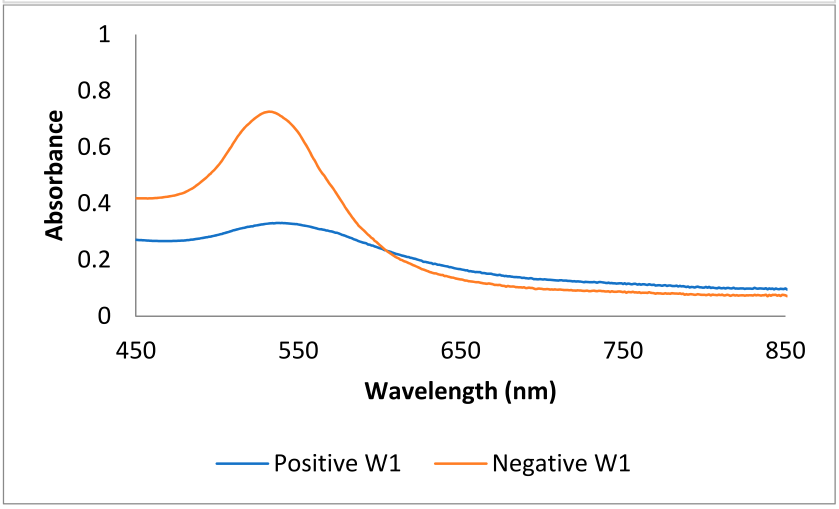

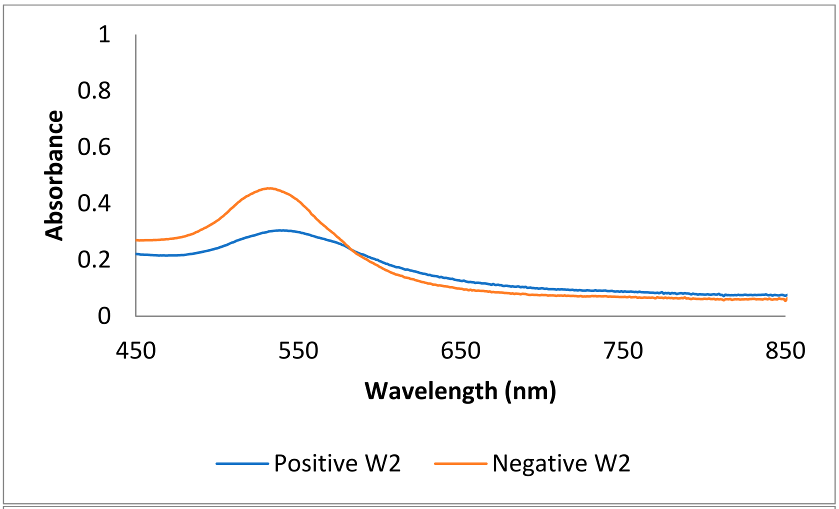

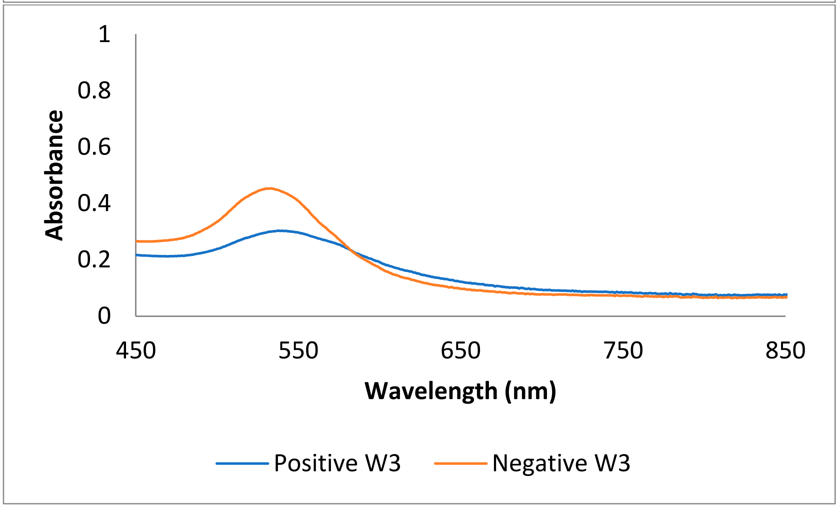

(B)

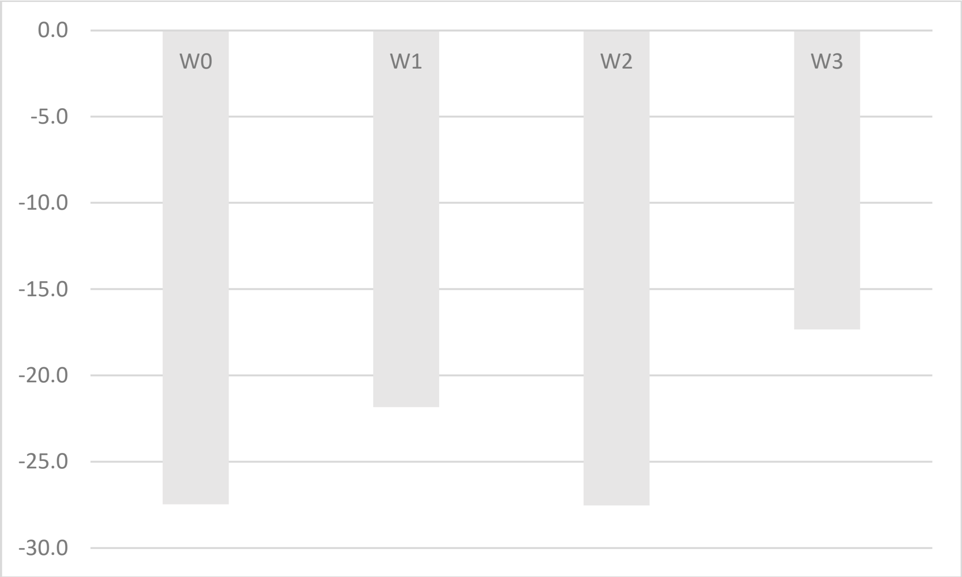

Figure S15. – Stability assay. AuNPs GRA6II-BSA storage at 4ºC during 3 weeks. UV-Vis spectrum in the presence of positive and negative samples after 1 week (W1), 2 weeks (W2) , and 3 weeks (W3) of storage (A).AuNPs GRA6II zeta potential measured immediately after nanoconjugation (W0), and after 1 week (W1), 2 weeks (W2) and 3 weeks (W3) of storage at 4ºC (B).
